# Unexpected Pericardial Cytology in a Patient With Primary Squamous Cell Carcinoma of the Lung: Emphasizing the Need for Advanced Molecular Characterization of Tumors

**DOI:** 10.7759/cureus.116243

**Published:** 2026-09-14

**Authors:** Melinda Gevorgian, Alex Minter

**Affiliations:** 1 Radiology, Alabama College of Osteopathic Medicine, Dothan, USA; 2 Hematology and Oncology, UAB (University of Alabama at Birmingham) Medicine – Russell Medical Cancer Center, Alexander City, USA

**Keywords:** adenosquamous carcinoma, cytologic-histologic discordance, non-small cell lung cancer (nsclc), pd-l1 expression, pericardial effusion, tumor heterogeneity

## Abstract

Non-small cell lung cancer (NSCLC) encompasses a diverse group of histologic subtypes, including adenocarcinoma, squamous cell carcinoma (SqCC), and the less common but clinically aggressive adenosquamous carcinoma. Intratumoral histologic heterogeneity is a recognized challenge in NSCLC and may result in diagnostic discordance when sampling is limited. Cytologic-pathologic discordance may raise concern for tumor heterogeneity, sampling error, or misidentified histologic subtype. In such scenarios, molecular profiling and re-biopsy are essential to refine diagnosis and guide therapy.

Here, we present a case of a 70-year-old man with a known history of SqCC of the lung who presented with progressive shortness of breath and was found to have a large pericardial effusion. Cytologic analysis of the pericardial fluid revealed malignant adenocarcinoma cells, discordant with the prior SqCC diagnosis. Molecular profiling of the pericardial fluid identified a TP53 mutation, intermediate tumor mutational burden, and microsatellite stability, without actionable oncogenic drivers. Repeat tissue sampling reconfirmed SqCC histology with high PD-L1 expression. The patient was initially treated with pembrolizumab monotherapy but required escalation to carboplatin, pemetrexed, and pembrolizumab due to disease progression. The cytologic discordance raised suspicion for unrecognized adenosquamous carcinoma or tumor heterogeneity not captured on initial biopsy. No evidence of a second primary malignancy or histologic transformation was found. This case highlights the diagnostic challenges and therapeutic implications of cytologic-pathologic discordance in NSCLC. It underscores the importance of molecular profiling and repeat tissue sampling in guiding personalized treatment decisions when histologic uncertainty exists.

## Introduction

Histologic classification of non-small cell lung cancer (NSCLC) can be challenging when based on small biopsy or cytology specimens that may not capture the tumor’s full architecture or histologic diversity [1]. Effusion cytology presents additional challenges because tissue architecture is absent and reactive mesothelial cells, poorly differentiated carcinoma, and different NSCLC subtypes may exhibit overlapping morphologic features [2,3]. Consequently, lineage-specific immunostaining and adequate tissue sampling may be necessary to distinguish squamous from glandular differentiation. This distinction has therapeutic implications because histologic subtype influences systemic treatment selection, including the choice of cytotoxic agents and interpretation of biomarker findings [4,5]. Adenosquamous carcinoma is a rare and aggressive NSCLC subtype defined by adenocarcinoma and squamous cell carcinoma components, each comprising at least 10% of the tumor [6]. Because limited specimens may sample only one component, this diagnosis generally requires evaluation of adequately sampled tissue. Pericardial effusions are more commonly associated with adenocarcinoma than squamous cell carcinoma [7]. We present a patient with tissue-confirmed squamous cell carcinoma whose subsequent pericardial-fluid cytology was interpreted morphologically as consistent with adenocarcinoma, illustrating the diagnostic and therapeutic uncertainty that can arise from discordant limited specimens.

## Case presentation

A 70-year-old man with a history of nicotine dependence, chronic obstructive pulmonary disease, and squamous cell carcinoma (SqCC) of the left lung, the American Joint Committee on Cancer (AJCC) Stage T1b N0 M0 (stage group IA2) [8], was initially treated with stereotactic body radiation therapy (SBRT) to a total dose of 5250 cGy in 5 fractions. He lived independently and had no known family history of malignancy. Initial imaging identified a left lower lobe pulmonary nodule with mild fluorodeoxyglucose (FDG) uptake on PET scan, which was later confirmed to be SqCC via CT-guided biopsy. Brain MRI showed no evidence of intracranial metastasis.

Approximately one year after completing radiation therapy, the patient developed progressive shortness of breath. Transthoracic echocardiography revealed a large pericardial effusion, and urgent pericardiocentesis yielded 1,200 mL of hemorrhagic fluid. Cytologic analysis reportedly demonstrated malignant epithelial cells interpreted morphologically as consistent with adenocarcinoma (Figure 1), a finding discordant with the patient’s prior tissue diagnosis of SqCC. Detailed cytomorphologic criteria and lineage-specific immunocytochemical results were not available for review; therefore, glandular differentiation and the site of origin could not be definitively established. Molecular profiling of the pericardial fluid (FoundationOne, Foundation Medicine, Cambridge, Massachusetts) identified a TP53 mutation, an intermediate tumor mutational burden of 10 mutations per megabase, and microsatellite stability. No actionable oncogenic drivers were identified.

**Figure 1:**
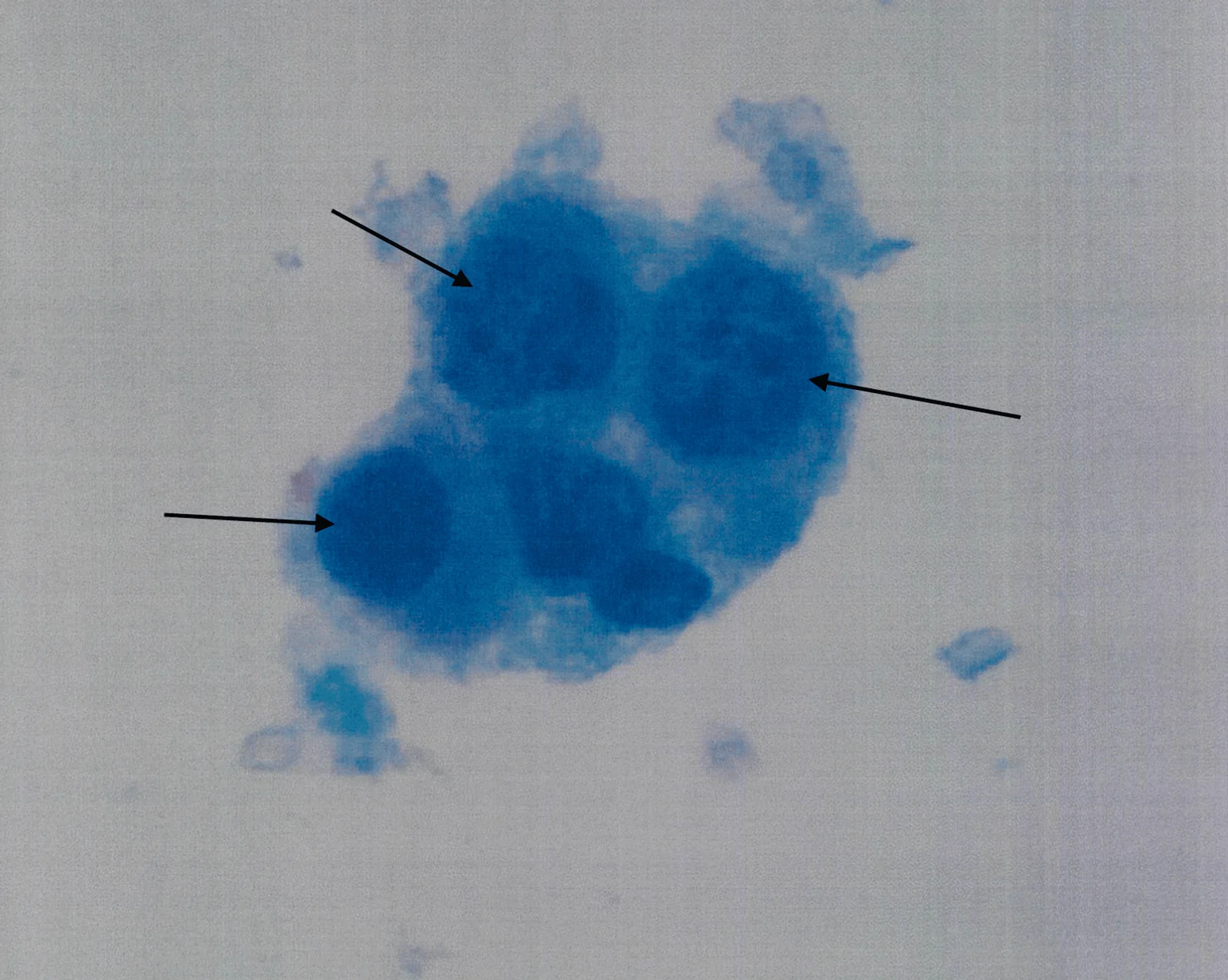
Cytologic evaluation of pericardial fluid demonstrating malignant epithelial cells interpreted morphologically as consistent with adenocarcinoma. Black arrows indicate representative atypical epithelial cells with enlarged hyperchromatic nuclei. Detailed cytomorphologic criteria and lineage-specific immunocytochemical results were not available for review.

Repeat tissue sampling by endobronchial ultrasound-guided biopsy was reported as consistent with squamous cell carcinoma. Detailed morphologic findings and lineage-specific immunohistochemical results supporting squamous differentiation were not available for review. PD-L1 immunohistochemistry demonstrated expression in 70% of tumor cells.

The patient was subsequently treated with pembrolizumab monotherapy. Following radiographic disease progression, manifested by enlargement of the left perihilar mass and development of bilateral pleural effusions, treatment was changed to carboplatin, pemetrexed, and pembrolizumab. The medical record available for review did not document the rationale for selecting a pemetrexed-containing regimen in the setting of tissue specimens interpreted as SqCC and pericardial-fluid cytology interpreted as adenocarcinoma. A 50% dose reduction in both chemotherapeutic agents was required during the second cycle because of treatment-related toxicity.

Although follow-up imaging demonstrated partial stabilization of disease in the lung and bones, the patient’s condition progressively declined. Despite supportive and palliative measures, he died several weeks later from complications of metastatic NSCLC. A complete timeline of the patient's diagnostic and therapeutic course is provided in Table 1, highlighting the interval between initial histologic diagnosis and discovery of discordant cytologic findings.

**Table 1:** Clinical timeline of disease progression, diagnostics, and treatment The timeline summarizes key diagnostic events, imaging findings, molecular results, and treatment decisions over a two-year course of care. PET, positron emission tomography; SUV, standardized uptake value; CT, computed tomography; MRI, magnetic resonance imaging; TNM, tumor-node-metastasis; SqCC, squamous cell carcinoma; SBRT, stereotactic body radiation therapy; cGy, centigray; TP53, tumor protein p53; TMB, tumor mutational burden; PD-L1, programmed death-ligand 1; EBUS, endobronchial ultrasound; IHC, immunohistochemistry; IV, intravenous.

Date	Event Description	Treatment/Intervention	Outcome/Progress
July 2022	PET scan: left lower lobe nodule (SUV 3.1)	—	—
October 2022	CT chest: Left lower lobe mass increased (1.2 cm to 1.8 cm)	—	—
November 2022	MRI brain: No metastatic disease. CT-guided biopsy: confirmed SqCC	—	—
January 2023	Completed definitive radiation therapy for Stage IA2, T1b N0 SqCC of the lung	SBRT; total dose of 5250 cGy in 5 fractions	—
February 2024	Progressive dyspnea. Large pericardial effusion detected on echocardiography	Pericardiocentesis performed	Cytology interpreted as consistent with adenocarcinoma cells
February 2024	Molecular testing of pericardial fluid	FoundationOne: TP53 mutation, TMB 10 mutations/megabase, microsatellite stable	No actionable oncogenic drivers
April 2024	Repeat tissue sampling to evaluate discordant cytology	EBUS-guided biopsy reported as SqCC; PD-L1 IHC performed on the repeat tissue specimen demonstrated expression in 70% of tumor cells	SqCC with PD-L1 expression in 70% of tumor cells
April 2024	Disease progression on imaging	Pembrolizumab 200 mg IV every 3 weeks	Initiated immunotherapy
October 2024	Interval enlargement of left perihilar mass, new pleural effusions	Carboplatin + Pemetrexed + Pembrolizumab	Disease stabilization, stable skeletal lesions

## Discussion

Lung cancer is increasingly recognized as a heterogeneous disease, both histologically and molecularly. As early as 1985, Roggli and colleagues demonstrated that nearly half of lung tumors exhibited multiple histologic patterns, underscoring the challenge of achieving diagnostic accuracy using small biopsy specimens [1].

In this case, the identification of malignant epithelial cells interpreted morphologically as consistent with adenocarcinoma in the pericardial fluid, despite two tissue diagnoses of squamous cell carcinoma, raised several possible explanations. One consideration was adenosquamous carcinoma, a rare and aggressive subtype of NSCLC containing both glandular and squamous components [6]. Limited biopsy specimens could conceivably have sampled only a squamous component while missing coexisting glandular differentiation. However, adenosquamous carcinoma was not established because both histologic components were not demonstrated in an adequately sampled tissue specimen. Other considerations included sampling error or spatial heterogeneity, a separate pulmonary adenocarcinoma, metastatic adenocarcinoma from an extrapulmonary primary, treatment-associated morphologic alteration, and histologic transformation. None of these explanations could be definitively confirmed or excluded.

Immunocytochemical results from the pericardial-fluid specimen, including lineage-specific markers such as TTF-1, Napsin A, p40, CK5/6, CK7, and BerEP4, were not available for review. Therefore, the reported cytologic interpretation of adenocarcinoma could not be independently confirmed. This limitation made it difficult to distinguish glandular differentiation from potential mimics, including reactive mesothelial cells and poorly differentiated carcinoma. Furthermore, the TP53 mutation and intermediate tumor mutational burden identified in the pericardial-fluid specimen were nonspecific and did not establish tumor lineage, intratumoral heterogeneity, or a clonal relationship with the tissue-confirmed squamous cell carcinoma. Because matched molecular profiling of the tissue and fluid specimens was unavailable, their relationship could not be determined.

The tumor’s high PD-L1 expression supported the inclusion of immune-checkpoint inhibition in the systemic treatment strategy [9]. However, the selection of a pemetrexed-containing regimen occurred in the setting of unresolved histologic discordance because pemetrexed-based chemotherapy is conventionally used for nonsquamous NSCLC [10-12]. If the treating clinician’s rationale was not documented, no definitive rationale for regimen selection can be inferred from the available records.

An important limitation of this retrospective case report is the availability of clinical and diagnostic detail from the patient’s original evaluation. More detailed vital signs and physical examination findings at presentation, including findings relevant to hemodynamic compromise or cardiac tamponade, were not available for review. Similarly, detailed imaging characteristics, cytomorphologic criteria, and lineage-specific immunocytochemical findings from the pericardial-fluid specimen were unavailable. These limitations restrict our ability to fully characterize the patient’s presentation and independently evaluate the reported cytologic diagnosis, and may reduce the reproducibility and educational value of the case for clinicians encountering similar presentations.

This case underscores the importance of reconsidering an initial diagnosis when cytologic findings are discordant with established tissue histology. When feasible, repeat tissue sampling, lineage-specific immunostaining, and comparative molecular profiling may help distinguish mixed histology or spatial heterogeneity from a separate primary malignancy, morphologic mimics, or histologic transformation. In this case, the underlying explanation for the discordance remained unresolved, emphasizing the importance of explicitly acknowledging diagnostic uncertainty when interpreting limited specimens and making treatment decisions.

## Conclusions

This case illustrates the diagnostic uncertainty that may arise when cytologic findings are discordant with established tissue histology. When feasible, repeat tissue sampling, lineage-specific immunostaining, and comparative molecular profiling may help clarify the cause of such discordance. When the findings cannot be definitively reconciled, the remaining uncertainty should be acknowledged when interpreting the diagnosis and selecting treatment.
